# Molecular mechanisms of neutrophil regulatory network in anti-Candida infection

**DOI:** 10.3389/fimmu.2025.1716645

**Published:** 2025-11-18

**Authors:** Yanhua Xu, Renyi Cheng, Wen Li, Wenjing Yu, Chider Chen

**Affiliations:** 1 Department of Orthodontics, Affiliated Stomatology Hospital of Kunming Medical University, Kunming, Yunnan, China; 2 Yunnan Key Laboratory of Stomatology, Kunming, China; 3 Department of Oral & Maxillofacial Surgery & Pharmacology, School of Dental Medicine, University of Pennsylvania, Philadelphia, PA, United States; 4 The Affiliated Hospital of Stomatology, School of Stomatology, Zhejiang University School of Medicine, and Key Laboratory of Oral Biomedical Research of Zhejiang Province, Hangzhou, Zhejiang, China; 5 Department of Orthodontics, School of Dental Medicine, University of Pennsylvania, Philadelphia, PA, United States; 6 Center of Innovation and Precision Dentistry, School of Dental Medicine, School of Engineering and Applied Sciences, University of Pennsylvania, Philadelphia, PA, United States

**Keywords:** neutrophil, oral candidiasis, Candida albicans, neutrophil extracellular trap (NET), immune regulatory networks

## Abstract

Candida albicans resides as a commensal within the oral mucosa but becomes pathogenic when epithelial or immune equilibrium collapses. Neutrophils constitute the dominant effector population controlling this transition, integrating pathogen- and host-derived cues into a coordinated antimicrobial program. Fungal β-glucan recognition through Dectin-1 and complement receptor 3 (CR3) activates spleen tyrosine kinase (SYK)–phosphoinositide 3-kinase (PI3K)–extracellular signal-regulated kinase (ERK) pathways and drives microtubule-associated protein 1 light chain 3B-II (LC3B-II) accumulation and NOD-like receptor family pyrin domain-containing 3 (NLRP3) inflammasome assembly, thereby coupling phagocytosis with oxidative burst and neutrophil extracellular trap (NET) formation. Caspase recruitment domain-containing protein 9 (CARD9)-dependent interleukin (IL)-17 and tumor necrosis factor-α (TNF-α) circuits sustain chemokine (C-X-C motif) ligand 1/2 (CXCL1/2)-directed neutrophil recruitment and granulocyte colony-stimulating factor (G-CSF)-mediated granulopoiesis, while tissue matrix components determine site-specific antigen handling and NETosis thresholds. Hypha-restricted peptide toxin Candidalysin links epithelial injury to NLRP3 activation and release of IL-1β, IL-6, and G-CSF, establishing an oropharyngeal candidiasis (OPC)-specific neutrophil regulatory loop critical for pathogen clearance but also for mucosal inflammation. Conversely, fungal morphogenesis, biofilm organization, and metabolic rewiring dampen reactive oxygen species (ROS) generation and promote immune tolerance and drug resistance. Clinically, G-/granulocyte-macrophage colony-stimulating factor (GM-CSF) adjuvants and G-CSF-mobilized granulocyte transfusion offer context-dependent benefits yet pose toxicity risks, underscoring the need for precise intervention in neutrophil activation. Advances in single-cell and spatial multi-omics approaches are uncovering the metabolic and functional heterogeneity of neutrophils within mucosal environments, providing mechanistic insight for targeted immunomodulation.

## 1 Introduction

Oral candidiasis is one of the most common human fungal infections, primarily caused by the opportunistic pathogen Candida albicans. Studies have shown that in immunocompetent populations, approximately 45% may be colonized by Candida albicans, while in immunocompromised patients, such as HIV-infected individuals and organ transplant recipients, the risk of infection significantly increases (1). More importantly, oral infections can become a crucial source of deep and disseminated infections, particularly in severely immunocompromised patients (2).

As one of the most important effector cells in the innate immune system, neutrophils play a vital role in resisting Candida infections. Research indicates that deficiencies in neutrophil count or function are closely associated with infection occurrence and prognosis (3). Recent studies reveal that neutrophils participate in anti-Candida immune responses through multiple mechanisms, including phagocytosis, release of reactive oxygen species (ROS), and formation of neutrophil extracellular traps (NETs) (4, 5).

Notably, neutrophil activation and function exhibit distinct tissue specificity. Studies have found that at different infection sites, neutrophils are recruited and activated through specific signaling pathways and display unique functional characteristics (6–10). However, Candida albicans has evolved complex immune evasion strategies. Studies show that clinical isolates can interfere with neutrophil function through various mechanisms, including biofilm formation, morphological transition regulation, and secretion of specific factors (11–14). Particularly in biofilm-associated infections, Candida not only demonstrates enhanced immune evasion capabilities but often exhibits significant antifungal drug resistance. Recent research has also discovered that Candida albicans can influence neutrophil metabolic reprogramming, thereby interfering with their immune function (15).

Neutrophil responses to Candida albicans are dynamic and highly context dependent, particularly within the oral mucosa, where epithelial integrity, microbial composition, and local immune tone collectively shape host resistance. Acting as both indispensable effectors and potential mediators of tissue injury, neutrophils mount rapid antifungal activity while at times amplifying inflammation and collateral damage. Recent evidence indicates that their roles extend beyond direct fungal killing to encompass metabolic coordination, antigen presentation, and reciprocal communication with epithelial cells that influence mucosal immune homeostasis (16, 17). Distinguishing protective from pathological neutrophil responses remains challenging because of spatial heterogeneity within mucosal tissues, the limited translational relevance of existing murine models, and the complexity of microbial–immune interactions (16). Increasing use of single-cell and spatial multi-omics approaches has begun to define context-specific neutrophil programs and their evolution during Candida infection (18, 19). At the same time, therapeutic strategies targeting neutrophil metabolism, reactive oxygen species production, or extracellular trap clearance are showing promise in enhancing antifungal efficacy while limiting tissue injury (20). Collectively, these advances provide a framework for developing precision immunotherapies that strengthen epithelial barrier defense and restore immune balance in oropharyngeal and other mucosal forms of candidiasis.

**Figure 1:**
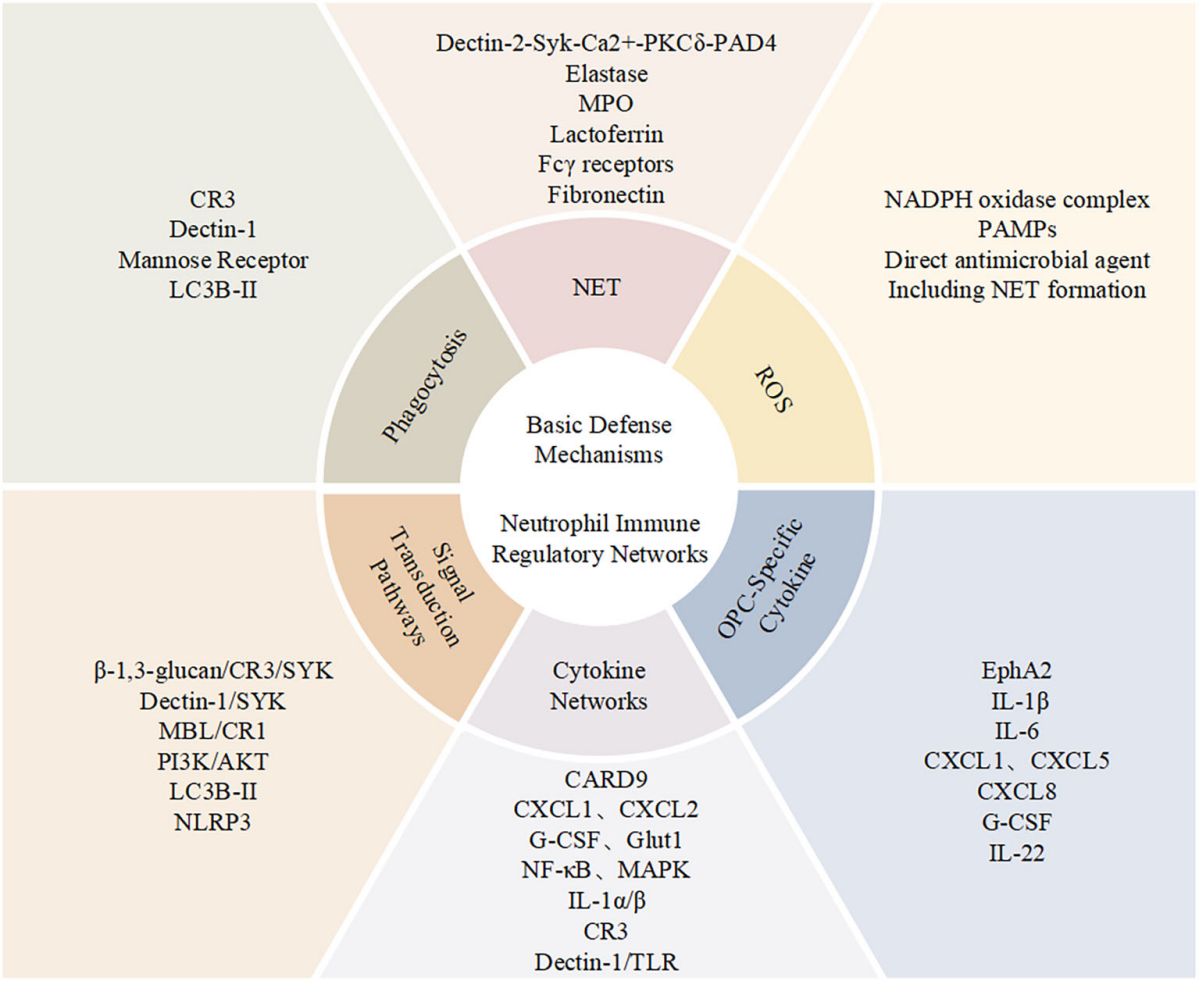
Neutrophil functional network in antifungal immunity. Schematic overview of neutrophil-mediated immune mechanisms during Candida infection, summarizing key molecular interactions, effector responses, and signaling pathways, together with a panel of OPC-related cytokines.

Elucidating the mechanisms of neutrophil function in anti-Candida infections is crucial for developing new therapeutic strategies. Currently, individualized treatment options for patients with immune dysfunction remain limited. With increasing drug resistance, traditional antifungal treatments face severe challenges (21). Developing immunomodulatory strategies targeting enhanced neutrophil function has become an important research direction in this field. This article will systematically review the progress in molecular mechanism research of neutrophil involvement in anti-Candida infections, focusing on their regulatory networks in different tissue microenvironments, and discuss the translational prospects of related research in clinical applications (**Figure 1**).

## 2 Basic defense mechanisms of neutrophils

Neutrophils are key innate immune cells in defending against Candida albicans infections, exerting antifungal effects through a complex and precise defense network. This defense system primarily comprises three major effector mechanisms: phagocytosis, neutrophil extracellular trap (NET) formation, and reactive oxygen species (ROS) production. These mechanisms work in coordination and close cooperation to form an important barrier against Candida infections (4, 22, 23).

In terms of phagocytosis, neutrophils rely on various pattern recognition receptors (PRRs) to recognize pathogen-associated molecular patterns of Candida albicans (24). Complement receptor 3 (CR3, consisting of CD11b/CD18) recognizes β-glucans through its unique lectin-like domain (25), Dectin-1 specifically recognizes β-1,3-glucans, while mannose receptors recognize cell wall mannan components (26). Neutrophil phagocytosis of Candida exhibits marked functional heterogeneity, encompassing at least two complementary effector programs. The first is a phagocytic–oxidative pathway coupled to the respiratory burst: upon activation, the NADPH oxidase complex assembles at the plasma and phagosomal membranes to generate superoxide, which is rapidly dismutated to H_2_O_2_; myeloperoxidase (MPO) then converts H_2_O_2_ to hypochlorous acid (HOCl) and, through reactions with amines, forms chloramines with additional microbicidal activity, thereby achieving potent oxidative killing within phagosomes and at adjacent extracellular sites (27–29). The second is a recently established extracellular mechanism—NETs: during the specialized program NETosis, chromatin decondenses and the nuclear and granule membranes are reorganized, culminating in the release of an expanded DNA–histone meshwork whose surface is decorated with microbicidal factors derived from granules and cytosol, including serine proteases and antimicrobial peptides (e.g., calprotectin). This enables the capture, confinement, and direct damage of both yeast and hyphal forms of Candida; NETs are particularly advantageous when pathogen burden is high, hyphae/biofilms are formed, or targets are poorly phagocytosed (30, 31). Functionally, NETosis corresponds to a “rapid-release” phenotype that establishes an immediate extracellular barrier within a short time, whereas the respiratory burst–phagocytic pathway provides sustained, primarily intracellular killing. Together, these programs coordinate across time and space to constitute a dual, complementary defense against Candida.

NET formation represents another crucial defense mechanism, with its structure composed of decondensed chromatin scaffolds and various antimicrobial proteins (such as elastase, MPO, and lactoferrin) (32). Studies have demonstrated two parallel but interconnected pathways for NET formation: NADPH oxidase-dependent and independent pathways. The former operates primarily through Fcγ receptors and protein kinase C (PKC), requiring ROS participation, while the latter proceeds through the Dectin-2-SYK-Ca2+-PKCδ-protein arginine deiminase 4 (PAD4) signaling cascade (33). Particularly noteworthy is the important regulatory role of extracellular matrix fibronectin (FN) in NET release, providing new insights into how tissue microenvironments modulate immune responses (34). Furthermore, hyphal forms of fungal filaments induce stronger NET release compared to yeast forms, reflecting the host immune system’s differential response to pathogens at various invasion stages (32).

The generation of reactive oxygen species (ROS) constitutes the third fundamental defense mechanism. Studies have demonstrated that neutrophils predominantly produce ROS through the activation of the NADPH oxidase complex, a process essential for antifungal immunity (35). Indeed, multiple stimuli can initiate NADPH oxidase complex activation, including phagocytic processes and the recognition of pathogen-associated molecular patterns (PAMPs) (36). Significantly, ROS demonstrates bifunctional properties in antifungal immunity: it serves both as a direct antimicrobial agent through oxidative damage and as a critical signaling molecule to regulate various immune processes, including NET formation (35, 37, 38).

## 3 Neutrophil immune regulatory networks

Candida albicans infection can initiate a multi-layered immune regulatory network: as central effectors, neutrophils integrate signals from Dectin-1/PRR-SYK, integrins, and complement/FcγR. Through canonical kinase and inflammasome pathways, neutrophils spatiotemporally coordinate the progression of phagocytosis, oxidative burst, and NETs. Meanwhile, cytokine networks including IL-17, IL-1, and TNF shape granulopoiesis, recruitment, and effector thresholds, ensuring precise defense against mucosal and invasive infections (39–42).

At the molecular level, these defense mechanisms are controlled by sophisticated regulatory networks. The β-1,3-glucan/CR3/SYK axis (39), similarly, the Dectin-1/SYK pathway (40), Mannan-binding lectin (MBL)/complement receptor 1 (CR1, CD35) signaling system (26) constitute essential molecular foundations of antifungal immunity, participating in Candida albicans recognition and signal transduction. These pathways regulate neutrophil functions, including phagocytosis, autophagy, and the production and regulation of ROS, through classical signaling cascades (such as PI3K/AKT and ERK (34)) and the newly discovered SYK-dependent LC3B-II accumulation mechanism (39). Furthermore, these molecular pathways activate key transcriptional regulatory pathways, such as NOD-like receptor signaling pathways, particularly the NLRP3 inflammasome (35), which further amplifies the immune response. These upstream signaling events and molecular regulation provide a solid foundation for the downstream cytokine network (26). Notably, these molecular recognition pathways exhibit distinct functional hierarchies in different infection contexts.

Neutrophil responses to Candida albicans differ markedly between systemic and mucosal tissues, shaped by distinct receptor repertoires and microenvironmental cues. In systemic candidiasis, recognition of fungal β-glucans by the C-type lectin receptor Dectin-1 (encoded by Clec7a) is pivotal for the activation of SYK–CARD9–NF-κB signaling, which induces pro-inflammatory cytokines such as TNF and IL-1β and promotes the generation of reactive oxygen species critical for fungal control (43, 44). Accordingly, Dectin-1-deficient mice are unable to restrict systemic fungal dissemination (45). In contrast, during oropharyngeal candidiasis, epithelial recognition of C. albicans depends primarily on EphA2- and TLR-driven pathways that coordinate early neutrophil recruitment (16). While Dectin-1 expression on resident oral macrophages contributes to immune signaling, its loss does not impair immediate neutrophil infiltration (46). At later stages, the adaptor CARD9 becomes indispensable for sustaining IL-17-mediated mucosal immunity and long-term protection. CARD9-deficient mice—and patients carrying deleterious CARD9 variants—exhibit susceptibility to chronic mucocutaneous candidiasis due to impaired Th17 cytokine responses and persistent fungal colonization (40, 47). These findings demonstrate that Dectin-1 governs early innate sensing of fungal β-glucans, whereas CARD9 serves as the essential signaling node linking pattern recognition to durable IL-17-dependent mucosal protection.

Molecular recognition and signal transduction processes often work in conjunction with cytokine networks to jointly amplify the effector functions of neutrophils in the inflammatory environment. Among these, the IL-17 signaling pathway is a core component of anti-Candida immunity, exhibiting multiple functions (41, 42). Candida albicans infection triggers Dectin-1 to activate dendritic cells and macrophages through caspase recruitment domain 9 (CARD9)-dependent signaling, which subsequently induces IL-17 production (48). The effects of IL-17 release regulatory signals through two major pathways: firstly, by inducing the secretion of chemokines such as C-X-C motif chemokine ligand 1 (CXCL1) and C-X-C motif chemokine ligand 2 (CXCL2) to form chemotactic gradients that recruit neutrophils to infection sites (49, 50); secondly, by promoting the production of granulocyte colony-stimulating factor (G-CSF) to enhance neutrophil generation and activation(32). Both genetic and acquired defects leading to IL-17 signaling abnormalities are directly associated with neutrophil dysfunction, representing one of the key pathogenic mechanisms for susceptibility to mucosal candidiasis (51). Beyond classical Th17-driven adaptive immunity, early innate IL-17 responses have emerged as key determinants of neutrophil recruitment during oropharyngeal candidiasis (OPC). In the oral mucosa, innate-like lymphocytes—including γδ T cells, natural Th17 cells, and group 3 innate lymphoid cells (ILC3s)—produce IL-17A and IL-17F within hours of C. albicans exposure, preceding conventional Th17 polarization (52, 53). These cells respond to epithelial-derived IL-1β and IL-23, stimulating G-CSF release and neutrophil-attracting chemokines CXCL1 and CXCL5 (54). Mice lacking γδ T cells or ILC3s exhibit delayed neutrophil infiltration and increased fungal burden (49, 55), highlighting the importance of the innate IL-17 axis in the early containment of infection. At the same time, epithelial sensing of pathogen-derived virulence factors provides an additional layer of neutrophil activation. Among these, the hypha-associated peptide toxin Candidalysin—encoded by ECE1—serves as a crucial epithelial trigger bridging fungal morphogenesis with host inflammation (56, 57). Upon hyphal contact, Candidalysin induces epithelial membrane damage and Ca²^+^ influx, activating EGFR–MAPK and NLRP3 inflammasome signaling, followed by IL-1β, IL-6, and G-CSF release that drives neutrophil recruitment (58, 59). In corticosteroid-induced OPC models, C. albicans ece1Δ/Δ mutants lacking Candidalysin fail to elicit these cytokines, resulting in impaired neutrophil infiltration and attenuated disease (56). Together, these findings establish Candidalysin as a central virulence determinant linking epithelial injury, cytokine induction, and neutrophil activity—thereby integrating fungal morphogenesis with innate and adaptive antifungal responses in the oral mucosa.

Furthermore, the TNF-α signaling pathway plays an equally important role in immune defense against Candida albicans (60). Following recognition of Candida albicans through pattern recognition receptors (such as Dectin-1), neutrophils release TNF-α, which acts on surrounding tissues through autocrine and paracrine mechanisms, activating NF-κB and mitogen-activated protein kinase (MAPK) signaling pathways. This further enhances neutrophil effector functions, including increased phagocytic rate, enhanced ROS production through NADPH oxidase, and stimulation of NET (neutrophil extracellular trap) formation (61). The TNF-α signaling pathway forms a synergistic network with the IL-17 pathway, jointly regulating neutrophil-driven antipathogen effector functions.

IL-1α and IL-1β from the IL-1 family also play crucial roles in neutrophil regulation. For instance, in early infection, Candida-stimulated keratinocytes release IL-1α, which stimulates local endothelial cells to secrete G-CSF, thereby establishing a “tissue-blood axis” between local tissues and bone marrow for rapid mobilization of neutrophil proliferation and migration. Additionally, IL-1-related signaling can precisely control neutrophil recruitment and directed migration through regulation of chemokine CXCL1/2 expression (62).

Moreover, some emerging cytokines further enhance neutrophil functions by working in conjunction with traditional mechanisms. For instance, IL-33 not only enhances phagocytic capacity by upregulating complement receptor CR3 expression but also increases neutrophil fungicidal activity through elevated ROS generation(41). IL-33 further modulates the Dectin-1/TLR signaling mechanism, promoting CXCL1/2 secretion, which strengthens neutrophil migration and activation(41). Animal experiments have demonstrated that IL-33 pretreatment significantly reduces Candida albicans infection-related mortality, indicating its potential clinical application value (63).

Beyond classic molecular recognition and cytokine networks, recent research has revealed novel regulatory mechanisms of neutrophils. For instance, NADPH oxidase not only serves as the primary pathway for ROS generation but also participates in more complex immune responses through signal regulation functions. Similarly, neutrophils’ adaptive adjustment of glucose metabolism plays a crucial supporting role in their efficacy. For example, the key role of glucose transporter-1 (Glut1) in neutrophil local energy metabolism contributes to efficient immune responses in the lesion microenvironment (26). These new functions integrate with classical receptor mechanisms (such as Dectin-1/SYK and CR3/SYK axes) to further enhance the flexibility and adaptability of neutrophils in anti-Candida immunity (39).

## 4 Current clinical treatment strategies

Current clinical treatment for Candida infections primarily encompasses two strategies: antifungal drug therapy and immune function modulation. Antifungal drugs mainly include azoles, echinocandins, and polyenes. Echinocandins (such as caspofungin), which inhibit β-1,3-D-glucan synthase in fungal cell walls, are the first-line treatment for invasive candidiasis in neutropenic patients (64, 65). For non-neutropenic patients, fluconazole remains the empirical treatment of choice due to its favorable bioavailability and safety profile (66). Polyene drugs (such as amphotericin B) are primarily used for severe and refractory infections, though their application is limited by significant nephrotoxicity (67).

However, antifungal therapy faces significant challenges. Clinical studies have shown that among patients with prolonged azole exposure and during hospital outbreaks—particularly those involving fluconazole−resistant Candida parapsilosis—clinical isolates often exhibit elevated resistance to fluconazole, itraconazole, and ketoconazole (68–70). Biofilm formation further compromises therapy by limiting drug penetration and conferring marked tolerance, leading to higher risks of treatment failure and relapse than in non−biofilm infections (71, 72). Mechanistically, Candida biofilms suppress neutrophil extracellular trap (NET) release and blunt ROS−dependent killing; these effects are conserved across clinical isolates with clear strain−dependent variation in ROS and NET responses (38, 73). In addition, clinically relevant drug–drug interactions and hepatorenal toxicities constrain antifungal selection and dosing, complicating management (71, 74).

Immune function modulation therapy primarily focuses on hematopoietic growth factor treatment and cytokine regulation (75, 76). G−CSF is the most commonly used immunomodulator in clinical practice, promoting neutrophil production and release while enhancing chemotaxis and phagocytic functions (77, 78). In neutropenic patients with invasive candidiasis, adjunctive use of G−CSF or G−CSF–mobilized granulocyte transfusions alongside antifungal therapy has been associated with improved clearance and survival, although the quality of evidence is low and recommendations are conditional (79, 80). Granulocyte−macrophage colony−stimulating factor (GM−CSF) has shown promising results as adjunctive therapy in refractory Candida infections by augmenting neutrophil phagocytic and fungicidal activity, including pilot and cohort studies of sargramostim in fluconazole−refractory disease and refractory invasive fungal infections (81–83).

Immune function modulation therapy primarily includes hematopoietic growth factor support and cytokine−pathway targeting; G−CSF, produced by endothelial and other stromal/immune cells, promotes neutrophil production and release while enhancing chemotaxis and phagocytic functions (80, 84). In invasive candidiasis with profound or prolonged neutropenia, clinicians may add G−CSF to antifungals to hasten neutrophil recovery, and consider GM−CSF (sargramostim) in selected refractory cases to augment phagocyte function; observational cohorts report improved responses with GM−CSF, although evidence quality remains low and use is case−selected (80, 85, 86). In practice, G−CSF (filgrastim) is administered by subcutaneous or intravenous injection once daily until a higher absolute neutrophil count (ANC) recovery, pegfilgrastim is given as a single subcutaneous dose in prophylaxis contexts, and GM−CSF (sargramostim) is typically delivered by subcutaneous injection in short adjunctive courses; in persistent, severe neutropenia, granulocyte transfusions from G−CSF–mobilized donors can serve as a short−term bridge while definitive antifungals and source control proceed (80, 84, 86). At mucosal surfaces, IL−17 and IL−22 cooperate to reduce oral fungal burden and enhance host defense by inducing epithelial antimicrobial programs, including S100A8/A9 and the chemokine CCL20, although contributions are context−dependent (53, 87, 88). IL−33 primes neutrophils for antifungal activity by tuning TLR and Dectin−1 signaling, promoting C-X-C motif chemokine receptor 2 (CXCR2)−axis chemokine responses, upregulating complement receptor 3 (CR3), and enhancing ROS−dependent killing in experimental models (63, 89, 90). These cytokines currently inform pathogenesis rather than routine therapy; recombinant IL−17, IL−22, or IL−33 are not established treatments for candidiasis outside research settings, so clinical immunomodulation in candidiasis relies on G−/GM−CSF alongside optimized antifungal therapy and source control (80, 88).

However, immunomodulatory therapy has limitations. Neutrophils can actively tune inflammation via neutrophil serine proteases (neutrophil elastase, proteinase−3, cathepsin G), which proteolytically process and can degrade pro−inflammatory cytokines such as interleukin-1 β (IL-1β) and tumor necrosis factor α (TNF-α); this immune “fine−tuning” helps explain inter−patient heterogeneity in responses to cytokine− or growth factor–based therapies (91, 92). Clinically, growth−factor–based immunotherapy is constrained by adverse effects: G−CSF commonly causes bone pain and leukocytosis; rare but serious complications include splenic rupture and capillary−leak phenomena, warranting careful dosing and monitoring, especially in patients at pulmonary risk (93, 94). GM−CSF (sargramostim) can induce flu−like symptoms, fever, edema, injection−site reactions, and leukocytosis, with dose−related increases in adverse events reported and risks detailed in regulatory labeling, including hypersensitivity and fluid retention in susceptible patients (95). More broadly, augmenting myeloid cytokine signaling (for example, with GM−CSF) can be a double−edged sword that skews inflammatory milieus and, context−dependently, may exacerbate disease processes, underscoring the need for individualized risk–benefit assessment and close clinical monitoring when using these agents as adjuncts to antifungal therapy (96). Recent research has revealed several important advances. The β-1,3-glucan/CR3/SYK pathway-dependent LC3B-II accumulation can enhance neutrophil fungicidal activity (97, 98). Single-cell sequencing has identified neutrophil subpopulations with distinct functional characteristics (17, 99, 100). Additionally, studies have confirmed that neutrophils upregulate glucose metabolism through selective expression of glucose transporter Glut1 to meet antifungal demands (40, 101, 102). These findings provide important clues for developing novel therapeutic strategies, although their clinical application value still requires further validation. Building on these advances, recent single-cell and spatial multi-omics investigations have provided unprecedented insight into the transcriptional and functional diversity of neutrophils residing in the oral and periodontal mucosa. These studies reveal that neutrophils are not a uniform antimicrobial population but instead encompass transcriptionally, metabolically, and developmentally distinct subsets that adapt to specific tissue niches and phases of inflammation.

## 5 Neutrophil heterogeneity and single-cell insights

Recent advances in single-cell and spatial multi-omics technologies have profoundly reshaped the current view of neutrophil heterogeneity in the oral mucosa (19). Rather than representing a uniform antimicrobial population, neutrophils display extensive transcriptional, metabolic, and functional diversity that reflects their adaptation to specific mucosal niches and inflammatory contexts. In human gingival tissue, single-cell RNA sequencing delineates at least two major neutrophil trajectories: (i) tissue-resident neutrophils, which persist under steady-state conditions and express high levels of survival and regulatory molecules such as BCL2A1 and IL1RN, relying primarily on oxidative phosphorylation; and (ii) inflammation-recruited neutrophils, characterized by enhanced glycolytic flux and elevated expression of NADPH oxidase subunits, enabling vigorous reactive oxygen species (ROS) production and robust NETosis (103). This metabolic divergence defines distinct effector thresholds—resident cells contribute to epithelial homeostasis through restrained ROS and NET release, whereas recruited neutrophils generate strong fungicidal activity at the expense of potential collateral tissue injury (103, 104). In chronically inflamed oral lesions, polymorphonuclear myeloid-derived suppressor cells (PMN-MDSC)–like cells with immunosuppressive transcriptional signatures (ARG1, S100A8/A9, PD-L1) have been identified, acting to curtail excessive inflammation and support tissue repair (105, 106). Trajectory analyses further indicate that emergency granulopoiesis driven by G-CSF signaling contributes to the emergence of these subsets, which exhibit metabolic and phenotypic plasticity—transitioning between antimicrobial and suppressive states in response to local cytokine and metabolic cues (105). The integration of single-cell transcriptomics with metabolic and spatial profiling thus reveals a continuum of neutrophil differentiation along a protective–regulatory–pathogenic axis. This multidimensional landscape provides a mechanistic basis for understanding how neutrophil diversity governs NETosis and ROS thresholds within oral tissues and informs precision therapeutic approaches that modulate neutrophil metabolism and differentiation to sustain antifungal defense while minimizing mucosal injury.

## 6 Conclusions and future directions

Neutrophils represent pivotal yet paradoxical regulators of oral mucosal immunity, acting as both essential effectors in antifungal defense and potential mediators of inflammatory injury. Recent findings demonstrate that their functions in Candida-associated infection extend beyond pathogen killing to encompass antigen presentation, metabolic cross−talk, and epithelial crosstalk that determine infection outcomes (107, 108). However, distinguishing protective from pathological neutrophil programs in the oral environment remains challenging due to spatial and temporal heterogeneity, limited model fidelity, and the complexity of local microbial–immune interactions (103). Integrative multi−omics and single−cell profiling are expected to delineate these context−dependent neutrophil trajectories (109, 110), while interventions targeting metabolic rewiring, ROS modulation, or NET clearance are emerging as strategies to enhance antifungal efficacy without exacerbating mucosal injury (111, 112). Translating these mechanistic insights into precise immunotherapies that reinforce barrier protection while restraining collateral inflammation will be crucial for future management of oral epithelial disease and periodontitis. Although considerable progress has been made in uncovering how neutrophil responses are regulated during Candida infection, important gaps remain when linking these findings to human biology. Much of our understanding comes from murine OPC models—most commonly cortisone-induced or Card9-deficient systems—that, while highly informative mechanistically, only partially recapitulate the architecture, microbiota, and immunoregulatory environment of the human oral mucosa (113, 114). Differences in epithelial keratinization, salivary composition, and microbial ecology all shape neutrophil recruitment and antifungal thresholds (115, 116). As a result, insights from animal studies regarding pattern-recognition receptor hierarchies (for example, limited Dectin-1 dependence), cytokine interactions (IL-17, IL-33), or metabolic remodeling may not fully reflect human physiology (117, 118).

In humans, OPC in otherwise healthy individuals is typically mild or self-resolving, suggesting a balanced relationship between C. albicans and epithelial-neutrophil homeostasis . Clinical disease tends to emerge under defined immunologic vulnerabilities—such as HIV infection, iatrogenic immunosuppression, or monogenic CARD9 deficiency—highlighting the challenge of extrapolating murine data to the wider population. Variation in fungal strain traits, epithelial signaling capacity, and the surrounding microbiome further complicates translation (119–121).

Bridging these differences will require experimental systems that more closely model human tissues, including three-dimensional oral mucosal co-cultures, microfluidic infection platforms, and longitudinal immune profiling in patient cohorts. Integrating these approaches with single-cell and spatial multi-omics analyses will be key to validating mechanisms defined in rodents and to developing precision strategies that harness neutrophil function against mucosal Candida infection (122–126).

## Glossary

AKT: Protein Kinase B
ANC: Absolute Neutrophil Count
CARD9: Caspase Recruitment Domain-containing Protein 9
CCL20: C-C Motif Chemokine Ligand 20
Clec7a: C-type Lectin Domain Family 7 Member A
CR1: Complement Receptor 1
CR3: Complement Receptor 3 (CD11b/CD18)
CXCL1: C-X-C Motif Chemokine Ligand 1
CXCL2: C-X-C Motif Chemokine Ligand 2
CXCL5: C-X-C Motif Chemokine Ligand 5
CXCR2: C-X-C Motif Chemokine Receptor 2
ECE1: Extent of Cell Elongation 1
EGFR: Epidermal Growth Factor Receptor
ERK: Extracellular Signal-regulated Kinase
G-CSF: Granulocyte Colony-stimulating Factor
Glut1: Glucose Transporter 1
GM-CSF: Granulocyte-macrophage Colony-stimulating Factor
H2O2: Hydrogen Peroxide
HIV: Human Immunodeficiency Virus
HOCl: Hypochlorous Acid
IL-1α: Interleukin-1 Alpha
IL-1β: Interleukin-1 Beta
IL-6: Interleukin-6
IL-17: Interleukin-17
IL-17A: Interleukin-17A
IL-17F: Interleukin-17F
IL-22: Interleukin-22
IL-23: Interleukin-23
IL-33: Interleukin-33
IL1RN: Interleukin-1 Receptor Antagonist
ILC3s: Group 3 Innate Lymphoid Cells
LC3B-II: Microtubule-associated Protein 1 Light Chain 3B-II
MAPK: Mitogen-activated Protein Kinase
MBL: Mannan-binding Lectin
MPO: Myeloperoxidase
NET: Neutrophil Extracellular Trap
NETosis: Neutrophil Extracellular Trap Formation
NF-κB: Nuclear Factor Kappa-light-chain-enhancer of Activated B Cells
NLRP3: NOD-like Receptor Family Pyrin Domain-containing 3
NOD: Nucleotide-binding Oligomerization Domain
OPC: Oropharyngeal Candidiasis
PAD4: Protein Arginine Deiminase 4
PAMPs: Pathogen-associated Molecular Patterns
PI3K: Phosphoinositide 3-kinase
PKC: Protein Kinase C
PKCδ: Protein Kinase C Delta
PRRs: Pattern Recognition Receptors
ROS: Reactive Oxygen Species
S100A8/A9: S100 Calcium-binding Protein A8/A9
SYK: Spleen Tyrosine Kinase
Th17: T Helper 17 Cells
TLR: Toll-like Receptor
TNF-α: Tumor Necrosis Factor-alpha.
